# Supplementary material for: Primary Care Service Provision Scale for Evaluating the Right to Health Among International Migrant Populations
Source: Healthcare (Basel). 2025 Aug 21;13(16):2068. doi: 10.3390/healthcare13162068 (PMC12385813; doi:10.3390/healthcare13162068)
Supplement: Supplementary file 1 [file healthcare-13-02068-s001.zip › healthcare-3724938-supplementary.pdf]

Table S1. The statistic calculation (56 items).

	Mean	D.S	A	K	Item-rest correlation	Item-rest correlation	Observation for the removal of ESEM items
					Escala	Dimension	
**Disp 1** The healthcare facility where I work has adequate physical infrastructure for the care we provide.	2.99	0.86	-0.61	-0.19	0.50	0.63	Factor loading ≥ 1
**Disp 2** The consultation rooms (boxes) at the healthcare facility where I work are adequate for the care we provide.	3.05	0.87	-0.63	-0.36	0.52	0.66	Factor loading < 0.3
**Disp 3** The number of consultation rooms at the healthcare facility where I work is adequate for the population we serve.	2.35	1.03	0.19	-1.11	0.50	0.63	Cross-loading on the 2nd factor ≥ 4
**Disp 4** The healthcare facility where I work has adequate waiting areas.	2.93	0.87	-0.41	-0.6	0.57	0.62	Cross-loading on the 2nd factor ≥ 4
**Disp 5** The healthcare facility where I work maintains adequate cleanliness of the premises and restrooms.	3.21	0.82	-0.79	-0.08	0.47	0.55	Factor loading < 0.3
**Disp 6** The healthcare facility where I work has signage that is understandable for international migrant populations.	2.6	0.94	-0.14	-0.86	0.41	0.38	Factor loading < 0.3
**Disp 7** The healthcare facility where I work provides all medications prescribed by the healthcare team	3.13	0.77	-0.72	0.31	0.52	0.49	Cross-loadings on the 2nd factor and a high residual correlation with another item.
**Disp 8** The healthcare facility where I work has the necessary equipment to carry out the indicated examinations.	3.13	0.75	-0.47	-0.34	0.51	0.59	Selected
**Disp 9** The healthcare facility where I work has modern equipment for patient care.	2.86	0.87	-0.43	-0.46	0.52	0.66	Selected
**Disp 10** The healthcare facility where I work has reasonable waiting times for care.	2.97	0.77	-0.37	-0.26	0.57	0.56	Cross-loading on the 2nd factor ≥ 4

**Disp 11** The healthcare facility where I work has available appointment slots for consultations with the various professionals.	2.62	0.82	-0.08	-0.54	0.53	0.61	Selected
**Disp 12** The healthcare facility where I work has sufficient staff to attend to patients.	2.58	0.92	-0.05	-0.83	0.50	0.58	Cross-loading on the 2nd factor ≥ 4
**Disp 13** The healthcare facility where I work has available dates and times for examinations.	2.81	0.74	-0.37	0.041	0.44	0.50	Cross-loading on the 2nd factor ≥ 4
**Disp 14** The healthcare facility where I work schedules appointments within a reasonable timeframe to conduct the requested examinations.	2.94	0.72	-0.38	0.06	0.50	0.52	Selected
**Disp 15** The healthcare facility where I work has clear processes for referring patients to a specialist or other professional when necessary.	3.06	0.74	-0.66	0.53	0.55	0.51	Cross-loading on the 2nd factor ≥ 4
**Disp 16** The healthcare facility where I work has staff who provide instructions and referrals that ensure continuity of care.	3.1	0.70	-0.70	0.92	0.57	0.56	Selected
**Disp 17** The healthcare facility where I work has someone who guides users at all times, especially migrants.	2.68	0.95	-0.26	-0.83	0.39	0.31	Factor loading <0.3
**Acep1** The healthcare facility where I work provides comprehensible information to users.	3.12	0.70	-0.48	0.10	0.66	0.62	Selected
**Acep2** The healthcare facility where I work has healthcare professionals who provide understandable, tailored information and instructions for the migrant population.	2.92	0.75	-0.32	-0.19	0.56	0.63	Factor loading <0.3
**Acep3** The healthcare facility where I work has administrative staff who provide understandable, tailored instructions for the migrant population.	2.82	0.79	-0.22	-0.43	0.59	0.62	Factor loading <0.3
**Acep4** The healthcare facility where I work has signage that is understandable and adapted for the migrant population.	2.5	0.85	-0.01	-0.60	0.49	0.59	Factor loading <0.3

**Acep5** The healthcare facility where I work has forms and documents that migrants must complete which are understandable and adapted for them.	2.58	0.84	0.02	-0.63	0.55	0.62	Selected
**Acep6** The healthcare facility where I work has forms and documents that migrants must complete in a language they can understand.	2.48	0.89	0.1	-0.73	0.53	0.58	Factor loading <0.3
**Acep7** The healthcare facility where I work has support materials (brochures) with understandable, adapted information for the migrant population.	2.43	0.90	0.171	-0.73	0.40	0.51	Factor loading <0.3
**Acep8** If a migrant person does not understand what they need to do, the healthcare facility where I work informs them where they can ask for assistance.	3.08	0.76	-0.70	0.48	0.57	0.59	Factor loading <0.3
**Acep9** T he healthcare facility where I work has an administrative team that is respectful of migrants' customs.	3.25	0.74	-0.65	-0.20	0.64	0.62	Selected
**Acep10** The healthcare facility where I work has healthcare professionals who take migrants' customs into account when providing instructions.	3.1	0.71	-0.43	-0.07	0.62	0.63	Selected
**Acep11** Regarding care for the international migrant population, the staff at the healthcare facility where I work listens attentively to migrant patients.	3.38	0.66	-0.73	-0.01	0.61	0.56	Factor loading ≥1
**Acep12** Regarding care for the international migrant population, the staff at the healthcare facility where I work treats patients well during their care.	3.46	0.63	-0.90	0.39	0.58	0.50	Factor loading <0.3
**Acep13** Regarding care for the international migrant population, the staff at the healthcare facility where I work treats migrants with respect.	3.56	0.59	-1.17	1.22	0.51	0.43	Factor loading <0.3
**Acep14** Regarding care for the international migrant population, the staff at the healthcare facility where I work respects patients' privacy at all times during care.	3.54	0.59	-0.98	0.41	0.545	0.46	Factor loading <0.3

**Acep15** The healthcare facility where I work has a healthcare team that is respectful of migrants' customs.	3.35	0.71	-0.86	0.37	0.56	0.59	Factor loading <0.3
**Ac1** The healthcare facility where I work is located close to where people need it.	3.56	0.56	-0.96	0.48	0.38	0.40	Factor loading <0.3
**Ac2** The healthcare facility where I work is located at a reasonable distance from the residences of the people it serves.	3.46	0.60	-0.71	-0.02	0.36	0.41	Factor loading <0.3
**Ac3** The healthcare facility where I work has public transportation that enables access at any time.	3.18	0.88	-0.92	0.12	0.21	0.23	r < 0.3 per dimension and overall scale.
**Ac4** The healthcare facility where I work is located in a safe area that allows users to access it easily at night or in the early morning.	2.6	1.01	-0.11	-1.07	0.35	0.3	Factor loading <0.3
**Ac5** The healthcare facility where I work has service hours that accommodate users' work schedules.	2.88	0.91	-0.38	-0.71	0.35	0.31	Selected
**Ac6** The healthcare facility where I work has appointment-request and service hours that ensure the safety of the population we serve.	2.99	0.78	-0.57	0.09	0.45	0.36	Factor loading <0.3
**Ac7** The cost (payment) for services at the healthcare facility where I work does not prevent migrant patients from using its services.	3.36	0.74	-1.03	0.66	0.46	0.47	Selected
**Ac8** The healthcare facility where I work allows migrant patients to access care from the professional they need without cost being an impediment.	3.47	0.70	-1.30	1.61	0.45	0.59	Factor loading <0.3
**Ac9** The healthcare facility where I work allows migrant patients to access examinations at the facility without cost being an impediment.	3.49	0.64	-1.14	1.34	0.43	0.61	Factor loading <0.3
**Ac10** The healthcare facility where I work allows migrant patients to access treatment without cost being an impediment.	3.47	0.64	-0.98	0.43	0.46	0.58	Selected

**Ac11** The healthcare facility where I work allows migrants to access the necessary healthcare even if they do not have the required documentation.	3.11	0.83	-0.64	-0.22	0.41	0.41	Factor loading <0.3
**Ac12** Regarding care for the international migrant population, the staff at the healthcare facility where I work treats migrant patients the same as Chilean patients.	3.44	0.69	-1.10	0.91	0.56	0.45	Selected
**Ac13** The healthcare facility where I work provides clear information on the administrative requirements (paperwork) that migrants must meet to receive care.	2.79	0.85	-0.15	-0.73	0.58	0.33	Factor loading <0.3
**Cal1** The healthcare facility where I work has staff with the necessary knowledge to answer questions about the health procedures they perform, especially for the migrant population.	3.05	0.81	-0.52	-0.29	0.59	0.51	Factor loading <0.3
**Cal2** Regarding care for the international migrant population, the staff at the healthcare facility where I work are adequately trained in migrant health.	2.83	0.90	-0.29	-0.75	0.49	0.43	Factor loading <0.3
**Cal3** Regarding care for the international migrant population, the staff at the healthcare facility where I work identify themselves at the moment of care.	3.22	0.72	-0.64	0.06	0.56	0.54	Factor loading <0.3
**Cal4** Regarding care for the international migrant population, the staff at the healthcare facility where I work are available and willing to answer any questions raised by migrant patients.	3.35	0.64	-0.69	0.37	0.58	0.62	Selected
**Cal5** Regarding care for the international migrant population, the physician/professional at the healthcare facility where I work dedicates sufficient time to perform the assessment and provide instructions	3.25	0.70	-0.66	0.16	0.55	0.68	Selected
**Cal6** Regarding care for the international migrant population, the administrative staff at the healthcare	3.23	0.69	-0.55	0.02	0.66	0.72	High residual correlation and overlap with other items.

facility where I work dedicates sufficient time to conduct assessments and provide instructions.							
**Cal7** Regarding care for the international migrant population, the staff at the healthcare facility where I work explains the procedure that will be carried out during the care provided.	3.39	0.63	-0.68	0.15	0.61	0.72	Selected
**Cal8** Regarding care for the international migrant population, the staff at the healthcare facility where I work clearly explains the available treatment alternatives.	3.35	0.65	-0.62	-0.03	0.63	0.75	High residual correlation and overlap with other items.
**Cal9** Regarding care for the international migrant population, the staff at the healthcare facility where I work explains the adverse effects of the prescribed medications or procedures.	3.19	0.77	-0.92	0.88	0.53	0.634	Selected
**Cal10** Regarding care for the international migrant population, the staff at the healthcare facility where I work provides clear and accurate diagnoses and instructions.	3.4	0.61	-0.66	0.29	0.62	0.75	Selected
**Cal11** Regarding care for international migrant populations, in the healthcare facility where I work, staff instill confidence and safety when performing procedures.	3.5	0.59	-0.88	0.70	0.64	0.71	Selected
